# Anti-proliferative and gene expression actions of resveratrol in breast cancer cells *in vitro*

**DOI:** 10.18632/oncotarget.2632

**Published:** 2014-11-08

**Authors:** Yu-Tang Chin, Meng-Ti Hsieh, Sheng-Huei Yang, Po-Wei Tsai, Shwu-Huey Wang, Ching-Chiung Wang, Yee-Shin Lee, Guei-Yun Cheng, Wei-Chun HuangFu, David London, Heng-Yuan Tang, Earl Fu, Yun Yen, Leroy F. Liu, Hung-Yun Lin, Paul J. Davis

**Affiliations:** ^1^ Taipei Cancer Center, Taipei Medical University, Taipei, Taiwan; ^2^ PhD Program for Cancer Biology and Drug Discovery, College of Medical Science and Technology, Taipei Medical University, Taipei, Taiwan; ^3^ School of Pharmacy, College of Pharmacy, Taipei Medical University, Taipei, Taiwan; ^4^ Core Facility, Taipei Medical University, Taipei, Taiwan; ^5^ Pharmaceutical Research Institute, Albany College of Pharmacy and Health Sciences, Albany, New York, USA; ^6^ Department of Periodontology, School of Dentistry, National Defense Medical Center and Tri-Service General Hospital, Taipei, Taiwan; ^7^ Department of Molecular Pharmacology, City of Hope National Medical Center and Beckman Research Center, Duarte, California, USA; ^8^ Albany Medical College, Albany, New York, USA

**Keywords:** stilbene, integrin αvβ3, apoptosis, anti-proliferation, breast cancer

## Abstract

We have used a perfusion bellows cell culture system to investigate resveratrolinduced anti-proliferation/apoptosis in a human estrogen receptor (ER)-negative breast cancer cell line (MDA-MB-231). Using an injection system to perfuse media with stilbene, we showed resveratrol (0.5 – 100 μM) to decrease cell proliferation in a concentration-dependent manner. Comparison of influx and medium efflux resveratrol concentrations revealed rapid disappearance of the stilbene, consistent with cell uptake and metabolism of the agent reported by others. Exposure of cells to 10 μM resveratrol for 4 h daily × 6 d inhibited cell proliferation by more than 60%. Variable extracellular acid-alkaline conditions (pH 6.8 – 8.6) affected basal cell proliferation rate, but did not alter anti-proliferation induced by resveratrol. Resveratrol-induced gene expression, including transcription of the most up-regulated genes and pro-apoptotic p53-dependent genes, was not affected by culture pH changes. The microarray findings in the context of induction of anti-proliferation with brief daily exposure of cells to resveratrol—and rapid disappearance of the compound in the perfusion system—are consistent with existence of an accessible initiation site for resveratrol actions on tumor cells, e.g., the cell surface receptor for resveratrol described on integrin αvβ3.

## INTRODUCTION

Resveratrol (trans-3, 4′, 5-trihydroxystilbene) is a naturally occurring polyphenol whose complex cellular actions are cell cycle arrest, differentiation, and apoptosis [1]. These cellular actions may reflect a reduction in intracellular reactive oxygen species (ROS) and mitochondrial trans-membrane potential Δ ψm, as well as reduced phosphorylation of mTOR, RP-S6 and 4EBP1 [2]. In addition, the compound is also reported to have anti-inflammatory, anti-leukemic, neuroprotective and antiviral properties [3–5]. A cell surface receptor for the stilbene has been identified on integrin αvβ3 and has been shown to be linked to activation of the MAPK signal transduction pathway [6]. The Arg-Gly-Asp (RGD) recognition site on the integrin blocks actions of the stilbene, indicating proximity of the resveratrol receptor to the RGD site [6, 7]. The mechanisms of anti-tumor effects of resveratrol are incompletely understood, but an end result in a variety of tumor cell models of resveratrol treatment is apoptosis [8–10].

Other beneficial effects attributed to resveratrol relate to aging, e.g., cataract and bone mass loss, and to neurodegeneration, obesity and diabetes [11]. Resveratrol induces expression of multiple genes and confers metabolic changes that mimic caloric restriction, a state which promotes longevity across species [12]. These benefits appear to be dependent upon sirtuin 1 (SIRT1), a NAD^+^-dependent deacetylase. How resveratrol activates SIRT1 protein is not entirely clear, but the binding of lamin A by SIRT1 induces an allosteric change in SIRT1 that exposes its deacetylase site to native substrates [11]. Modulation of SIRT1 activity is also dependent on physiological substrate sequence and these substrates may contribute to actions of resveratrol [13].

The pharmacokinetics of resveratrol have been studied in pre-clinical models and human subjects [14, 15] and appear unfavorable for application to clinical disease states [14]. The systemic bioavailability of resveratrol is poor, as is the case with many polyphenols. Studies in mice, rats and dogs consistently suggest that resveratrol is well-absorbed, but is avidly glucuronidated and sulfated in both liver and intestinal epithelial cells [16, 17]. Studies in human subjects also indicate poor bioavailability of unmodified resveratrol [18, 19]. Resveratrol has a very short half-life in the systemic circulation.

In the current studies, we have examined the anti-cancer properties of resveratrol in chemotherapy-resistant human breast cancer MDA-MB-231 cells, using an *in vitro* perfused cell model in which concentrations of the agent and durations of exposure to the agent can be critically adjusted. Ambient pH and medium composition are also readily manipulated and are important to tumor cell function [20]. Resistance of cancers to therapy can involve both biochemical and microenvironmental factors. Studies here include effects on resveratrol's activity of factors such as environmental pH of tumor cells, duration of cell exposure to resveratrol, and effectiveness of drug concentrations in terms of anti-proliferation. The microarray and real-time PCR studies are focused on genes whose expression is highly upregulated by resveratrol (such as *NLRP1*, *CASP2*, *LSM4* and *SLC12A4*) and genes that support apoptosis, e.g*., BAD, p53, TP53I3, p21, c-fos* and *COX-2*.

## RESULTS

### Effect of time of exposure on resveratrol-induced signal transduction, gene expression and anti-proliferation in MDA-MB-231 cells

**Figure 1:**
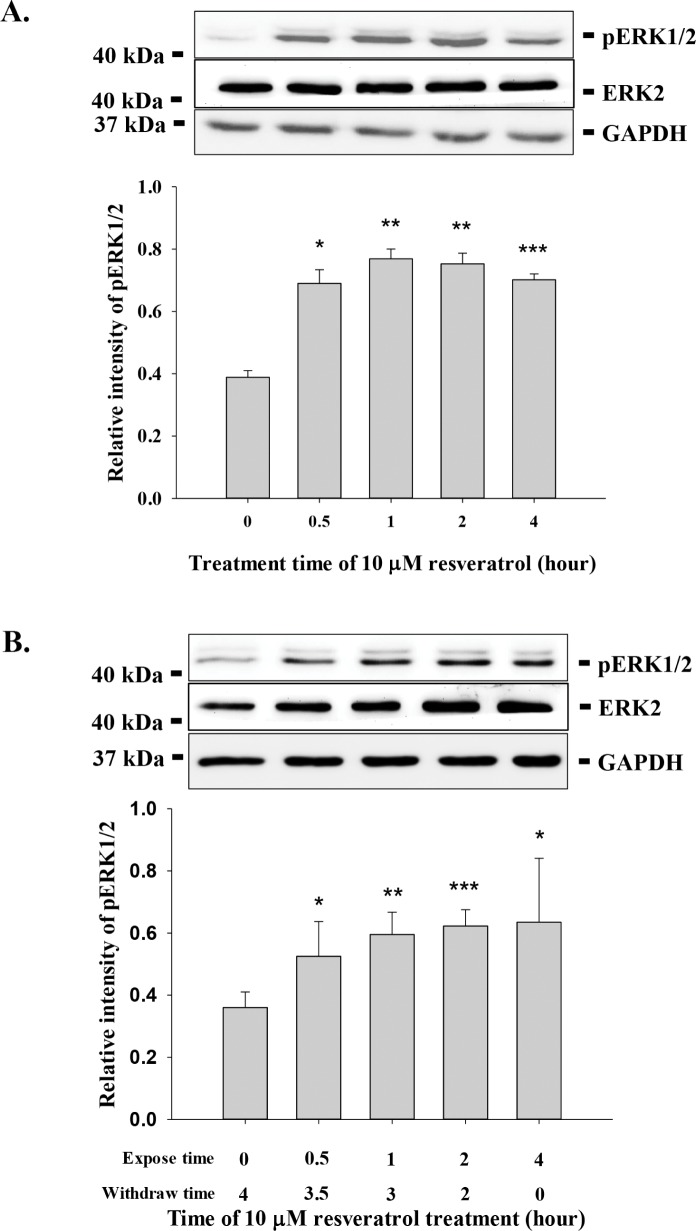

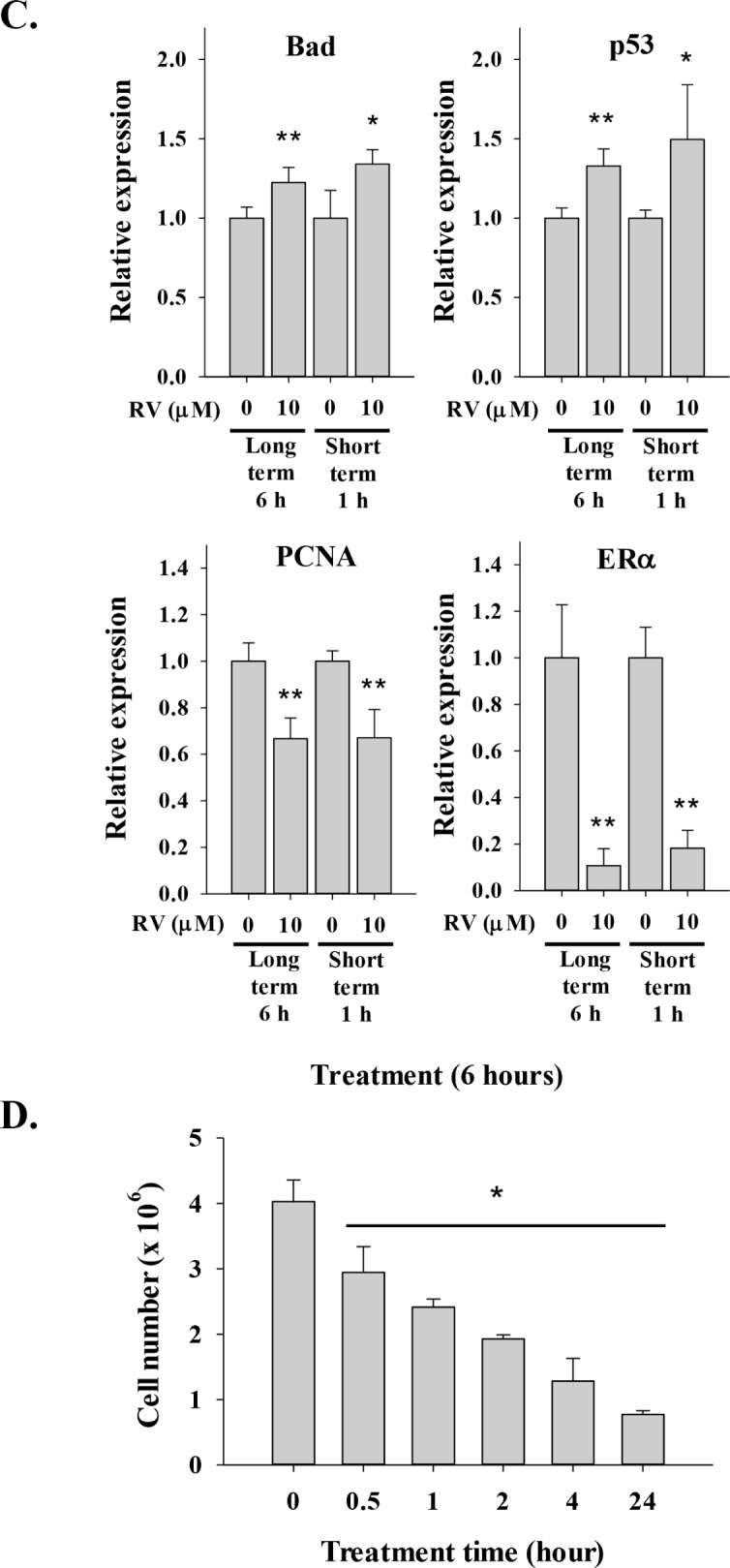
Incubation effect on resveratrol-induced signal transduction, gene expression and anti-proliferation **(A)** MDA-MB-231 cells were treated with 10 μM resveratrol for different time periods (0.5 to 4 h) prior to harvest. Cell extracts were separated by SDS-PAGE and blotted with anti-pERK1/2. Number of independent experiments (N) = 3. **(B)** MDA-MB-231 cells were treated with 10 μM resveratrol for different time periods (0.5 to 4 h) prior to removal of resveratrol and re-fed with fresh medium before harvest. Cell extracts were separated by SDS-PAGE and blotted with anti-pERK1/2 and anti-ERK2. N = 4. **(C)** MDA-MB-231 cells were treated with 10 μM resveratrol for different time periods (1 or 4 h) prior to removal of resveratrol and re-fed with fresh medium before harvest. Total RNA was extracted and qPCR was conducted as described. Number of independent experiments (N) = 3. **(D)** MDA-MB-231 cells were seeded in Petri dish and re-fed with DMEM containing 10% serum. 10 μM (228.24 × 10^−6^ mg/ml) of resveratrol was added to cell culture daily for 24, 4, 2, 1 and 0.5 h. After incubation, cells were re-fed with fresh medium. Cells were harvested at the 6th day and cell numbers were counted. N = 4 (*p < 0.05, compared to control with vehicle solvent)

Resveratrol has limited bioavailability, which has caused concern about its biological activity. Cellular uptake and metabolism of resveratrol is rapid [21]. To determine the minimum incubation time for resveratrol-activated signal transduction and anti-proliferation, MDA-MB-231 cells were treated with 10 μM resveratrol for 0.5 − 4 h prior to harvest. Exposure of cells for 0.5 h to resveratrol importantly activated ERK1/2 (Fig. 1A). To determine the duration of resveratrol-induced ERK1/2 activation after removal of the stilbene, we treated cells with resveratrol for 0.5 − 4 h, then removed the agent at specific time points. Cells were then washed twice with fresh medium before they were re-fed with medium for the remainder of the time prior to harvest. The activation of ERK1/2 exposed to resveratrol for 0.5 h did not decay as compared to that in the 4 h exposed cells (Fig. 1B).

The effect of duration of incubation with resveratrol on drug-modulated gene expression was determined by incubating cells with resveratrol for either 1 h or 6 h. The resveratrol was then removed and cells were washed twice with fresh medium and re-fed with fresh medium for another 5 h. There was no significant difference in resveratrol-upregulared and -downregulated gene expression with exposure to 10 μM resveratrol for 6 h and 1 h (Fig. 1C). When MDA-MB-231 cells were treated with 10 μM resveratrol for short periods of time (0.5 to 4 h) daily for 6 d, we found that exposure to the drug for 4 h reduced cell counts by more than 60%, compared to untreated control cells (1.28 × 10^6^ ± 3.46 × 10^5^ [resveratrol] vs. 4.03 × 10^6^ ± 3.29 × 10^5^ [control]). On the other hand, exposure of cells to 10 μM resveratrol for 24 h daily for 6 d caused 80% reduction in cell counts (7.72 × 10^5^ ± 5.44 × 10^4^) compared to untreated control (Fig. 1D). The full-term exposure to resveratrol increased only 20% more in anti-proliferative effect than those treated with 4 h daily. Those results suggest that short-term exposure to resveratrol is sufficient to induce cellular activities such as gene expression and anti-proliferation.

### Effect of acid-alkaline culture conditions on resveratrol-induced anti-proliferation in MDA-MB-231 cells

**Figure 2:**
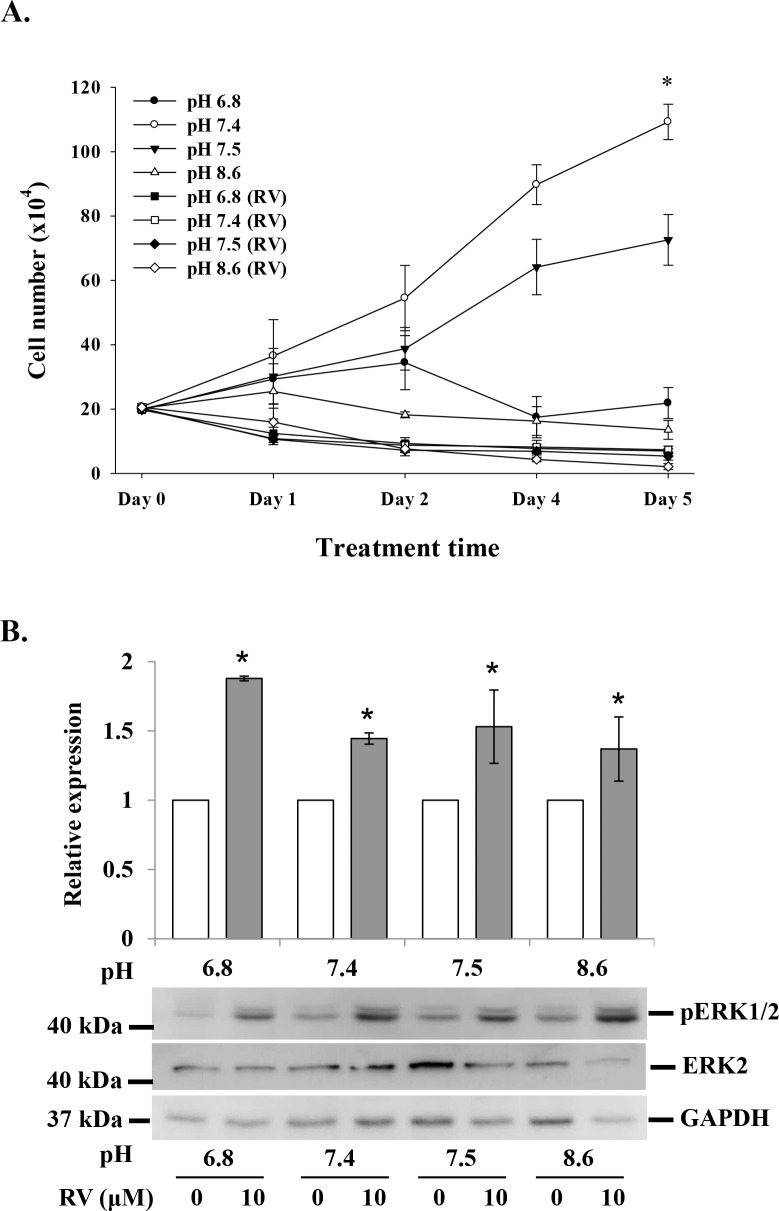
Effect of acid-alkaline culture conditions on resveratrol-induced anti-proliferation in MDA-MB-231 cells **(A)** MDA-MB-231 cells cultured in different pH conditions were treated with or without 10 μM resveratrol daily for 5 d. At the end of the culture period, cells were harvested and counted (N = 4). **(B)** MDA-MB-231 cells cultured in different pH conditions were treated with 10 μM resveratrol for 4 h. Cells were harvested and total protein was extracted and separated by SDS PAGE. pERK1/2 and ERK2 were evaluated. GAPDH was used as an internal control. N = 4 (*p < 0.05, compared to control with vehicle solvent)

It has been reported that the acidic condition of tumor microenvironment affects the efficacy of chemotherapy [20]. MDA-MB-231 cells were cultured in media with pHs of 6.8, 7.4, 7.5 and 8.6. Cell proliferation in the absence of resveratrol (10 μM) was significantly affected by pH culture condition, but the anti-proliferation effect of the stilbene was only minimally affected by pH (Fig. 2A). The resveratrol-induced activation of ERK1/2 was not affected by extracellular pH change (Fig. 2B).

### Microarray gene profiles in resveratrol-treated MDA-MB-231 cells

**Figure 3:**
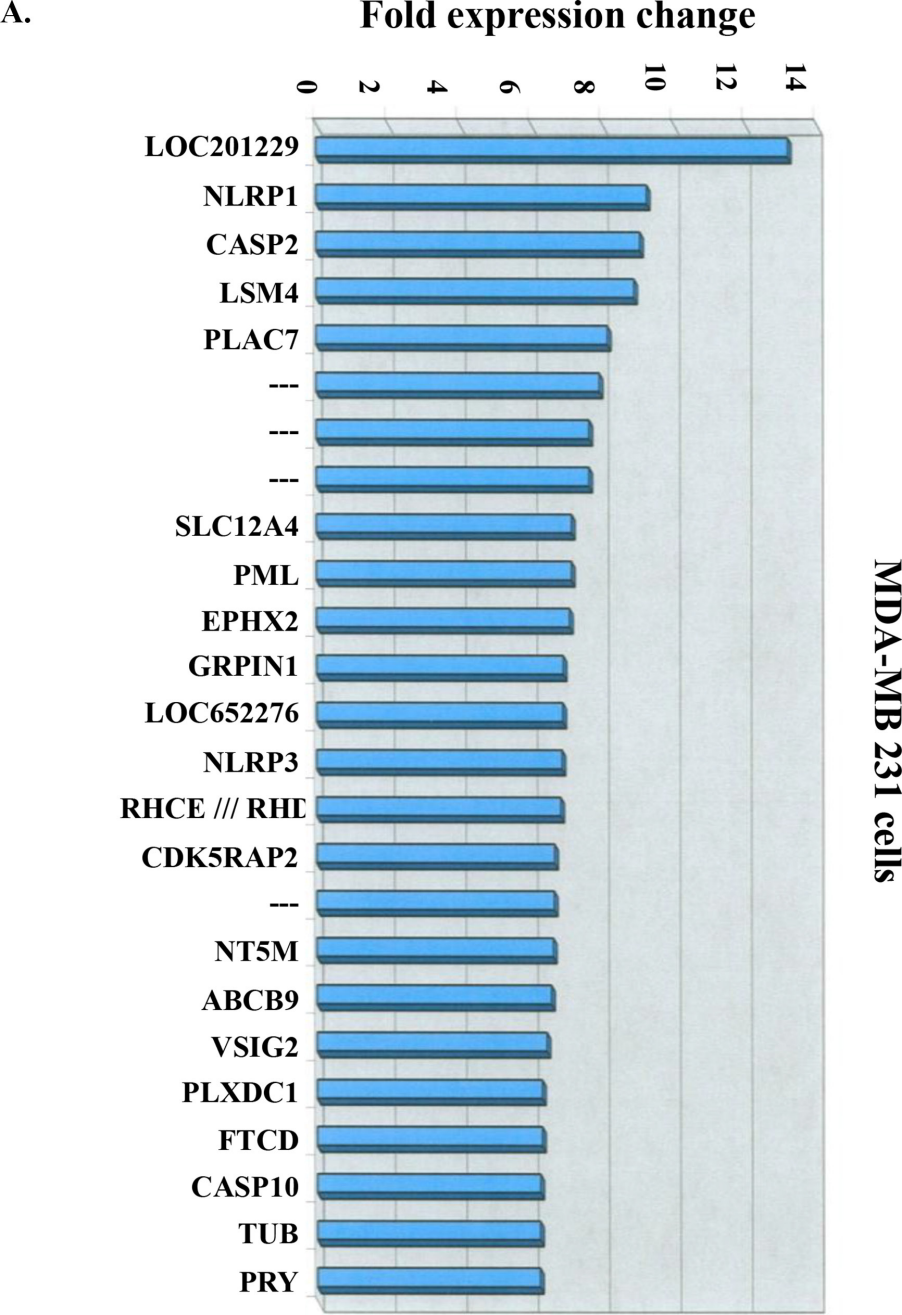

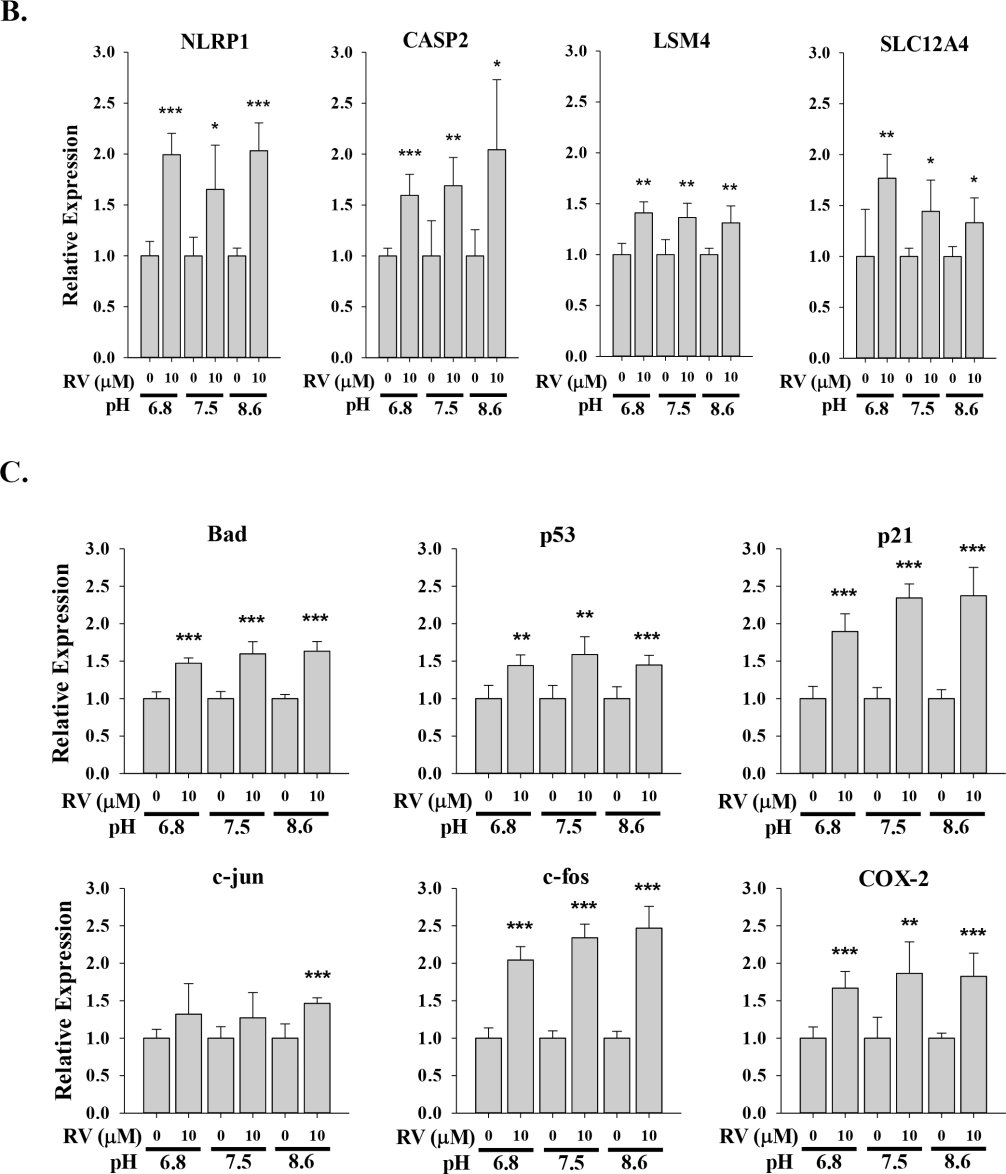
Resveratrol-induced gene expression is not affected by culture media pH **(A)** The most up-regulated genes induced by resveratrol in human breast cancer MDA-MB-231 cells. MDA-MB-231 cells were treated with or without 10 μM resveratrol for 6 h. Total RNA was harvested and hybrided with Affymetrix HG-U133 plus 2.0 microarray platform. 25 top up-regulated genes from 54675 probe sets. N = 2. **(B)** MDA-MB-231 cells cultured in different pH conditions were treated with 10 μM resveratrol for 6 h. Cells were harvested and total RNA was extracted for qPCR. Expression of 4 most up-regulated genes induced by resveratrol was examined. N = 4. **(C)** resveratrol-induced 6 p53-dependent genes were also examined. N = 3 (*p < 0.05; **p < 0.005; ***p < 0.001, compared to control with vehicle solvent)

MDA-MB-231 cells were exposed to 10 μM resveratrol for 6 h. Microarray experiments were conducted as described in the Materials and Methods and identified 25 highly up-regulated genes (≥6-fold increase in mRNA abundance) as major gene targets for resveratrol (Fig. 3A). The second most up-regulated mRNA with resveratrol treatment was encoded by the *NALP1* gene, also known as *NLRP1* and *DEFCAP*, an essential component of the inflammasome and known to play an important role in innate immunity. Recent hallmark reports clarify the role of this gene in caspase-1 activation and interleukin-1β production and in inflammasome assembly and function demonstrating that NALP1 is a direct sensor of bacterial components in host defense against pathogens [22]. The third most highly up-regulated mRNA was encoded by the *CASP2* (caspase 2) gene, a well-known apoptosis regulatory protein. It has been demonstrated by using *in vitro* coimmunoprecipitation experiments that NALP1 (DEFCAP) protein is capable of strongly interacting with caspase-2, and transient overexpression of full-length DEFCAP-L, but not DEFCAP-S, in breast adenocarcinoma cells MCF-7 resulted in significant levels of apoptosis [23]. Other up-regulated genes, such as *LSM4*, which is involved in RNA processing, may function in a chaperone-like manner [24]. *SLC12A4* (human potassium chloride cotransporter 1) is activated by cell swelling and may be associated with apoptosis [25, 26]. These results convey the anti-inflammation and anti-cancer properties of the stilbene. In order to further investigate whether environmental conditions affect the expression of those up-regulated genes induced by resveratrol, expression of *NALP1, CASP2, SLC12A4* and *LSM4* was studied.

MDA-MB-231 cells grown in different pH conditions were treated with 10 μM resveratrol for 6 h and total RNA was harvested for real-time PCR. Expression of *NALP1*, *CASP2*, *SLC12A4* and *LSM4* mRNAs were induced significantly under various conditions (Fig. 3B). We also studied pro-apoptotic protein abundance in resveratrol-treated cells harvested from the perfusion bellows system and demonstrated activation of several pro-apoptotic genes (Fig. 3C). Thus, resveratrol-induced gene expression and chemical/cellular activity are minimally affected by changes in the cancer microenvironment.

### Pharmacodynamic studies of resveratrol in the perfusion bellows cell culture system

**Figure 4:**
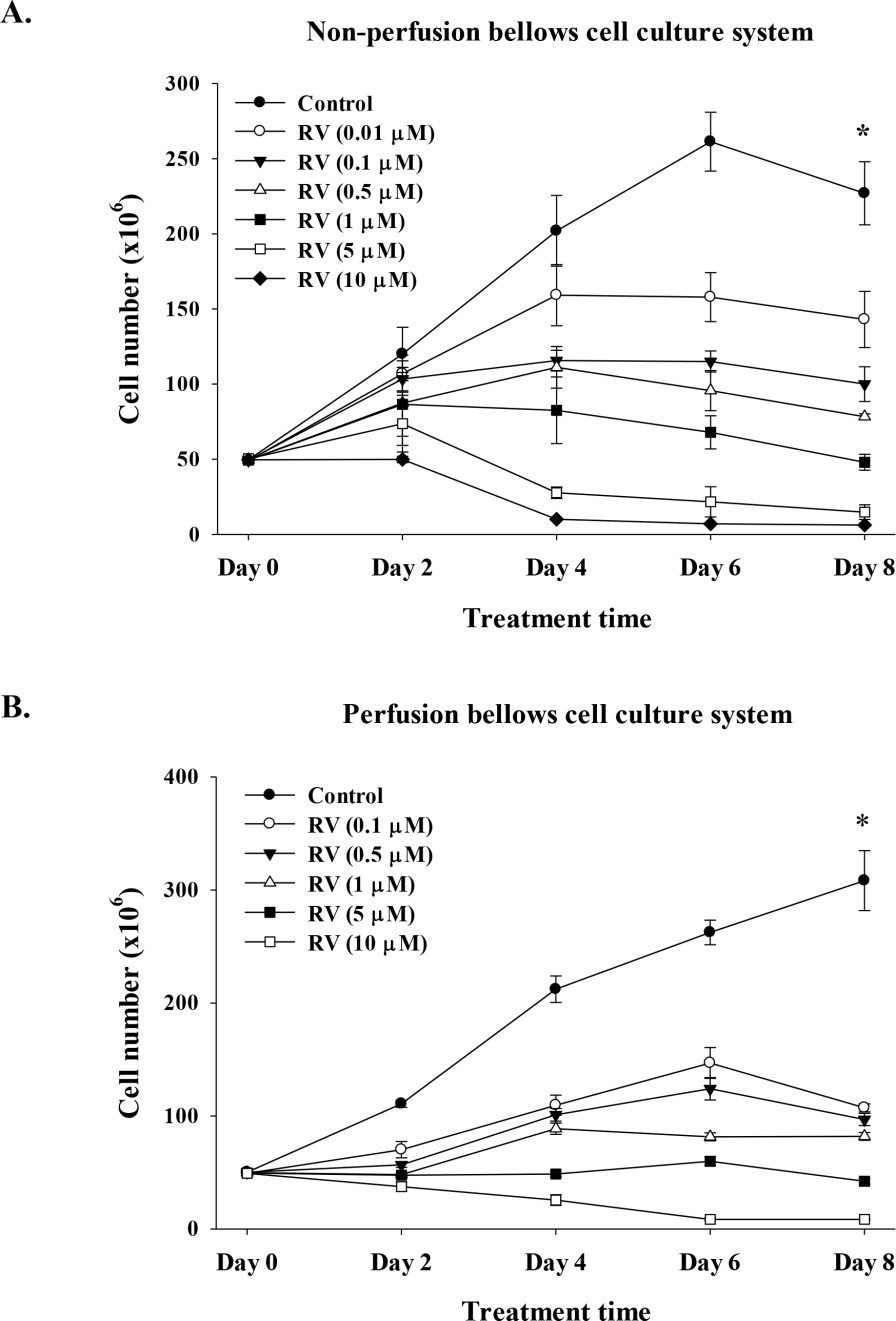

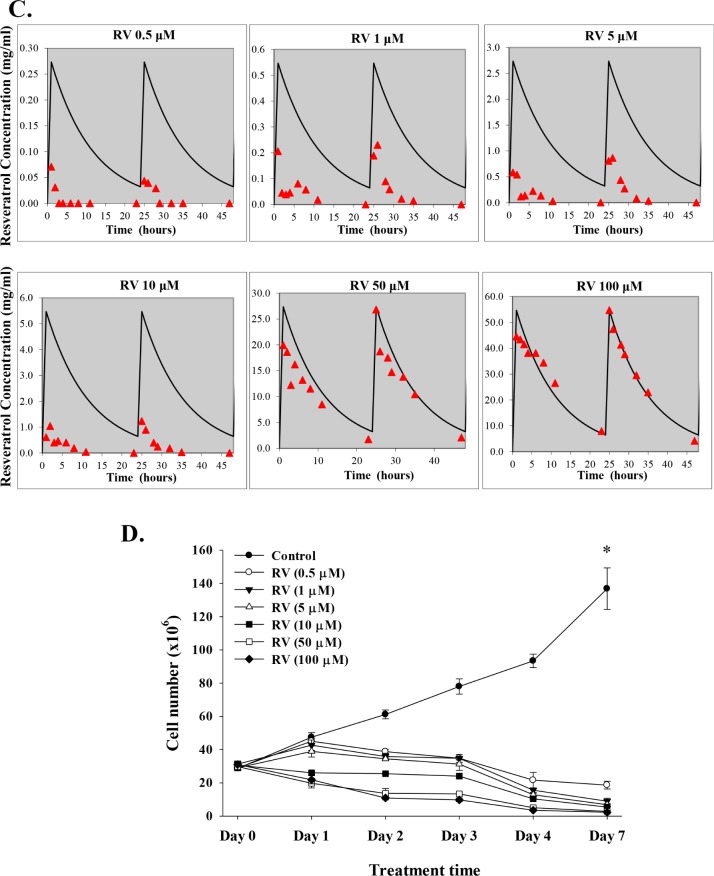
Resveratrol induces anti-proliferation in breast cancer MDA-MB-231 cells Cells were cultured in **(A)** non-perfusion **(B)** perfusion bellows cell culture system with 10% fetal bovine serum in culture medium for 8 d. Different concentrations (0.01 to 10 μM) of resveratrol were added in culture media daily, together with refreshed medium. Cells were harvested and cell numbers were counted every other day. Points, mean; bars, ± SD; N = 4 **(C)** Short-term exposure to resveratrol induces anti-proliferation of cancer cells in the perfusion bellows cell culture system. Determination of resveratrol concentration was performed by LC/MS/MS. Cells were cultured in the system with 10% fetal bovine serum in culture medium. Different concentrations of resveratrol were added in culture media daily by syringe pump in 1 h and no fresh resveratrol in influx medium for the another 23 h. One ml of medium was drawn from the efflux tube of the central reservoirs at different time points. Samples were stored at −20°C until examination. Concentrations of resveratrol were measured by LC/MS/MS as described in the Materials and Methods. N = 2. **(D)** Pharmacodynamic studies of resveratrol-induced anti-proliferation in perfusion bellows cell culture system. Cells were cultured in the perfusion bellows culture system with 10% fetal bovine serum in culture medium for 6 d. Different concentrations of resveratrol were added in culture media daily by syringe pump in 1 h and there was no fresh resveratrol in the influx medium for another 23 h; the final concentrations in the media were 0.5 to 100 μM. Cells were harvested and cell numbers were counted every day. N = 3 (*p < 0.05, compared to control with vehicle solvent)

MDA-MB-231 cells were seeded in the non-perfused bellows cell culture system, re-fed with medium containing 10% FBS after 24 h and refreshed daily thereafter. Cells were counted to confirm the numbers of cells initially attached to flakes. Resveratrol concentration in the medium was constant throughout each experiment and the resveratrol concentrations tested were 0.01 μM to 10 μM. An anti-proliferative effect of resveratrol was not detectable on cell counts at 2 d (Fig. 4A), regardless of stilbene concentration (up to 10 μM). Anti-proliferation was apparent by 4 d of treatment, at which time resveratrol caused a 1.3 to 20-fold (1.59 × 10^8^ ± 2.03 × 10^7^ to 1.01 × 10^7^ ± 2.75 × 10^5^) decrease in treated cells compared to unexposed control cells (2.02 × 10^8^ ± 2.36 × 10^7^). The resveratrol effect was concentration-dependent (Fig. 4A). In a second protocol, cells were exposed to 10% FBS-containing DMEM and treated with different concentrations of resveratrol (0.1 to 10 μM) daily for 8 d. Cells were then harvested from the perfusion bottles and counted. There were significantly different growth patterns of control cell cultures between perfusion and non-perfusion cell culture systems in extending the culture condition (Fig. 4B).

Because short-term exposure to resveratrol induced anti-proliferation in MDA-MB-231 cells (Fig. 1D), we determined whether short-term exposure to resveratrol induced anti-proliferation of cancer cells in the perfusion bellows cell culture system. MDA-MB-231 cells were cultured in cell culture system as described above. Different concentrations of resveratrol were injected into perfusion bellows bottles over a 1 h period and incubation continued for another 23 h without resveratrol in influx medium. We determined the concentrations of resveratrol harvested from perfusion bellows bottle by LCMS/MS. Media were harvested at various time points. The concentration of resveratrol in efflux tubes from bellows cell culture was far below the influx tube in the range between 0.5 μM to 100 μM (Fig. 4C). However, the concentrations of resveratrol in efflux tubes of the 50 μM and 100 μM input resveratrol concentrations followed the expected patterns (Fig. 4C). These results confirm previous reports that the turnover rate of resveratrol inside the cells is rapid. Interestingly, the results of 50 μM and 100 μM resveratrol experiments also suggest that resveratrol may not need to enter cells to induce apoptosis. For the anti-proliferation studies, the short-term 1 h-injection of resveratrol procedures were repeated for 6 d. Results presented in Fig. 4D indicate that although cells were exposed to resveratrol for a short period of time, resveratrol induced anti-proliferation.

**Figure 5:**
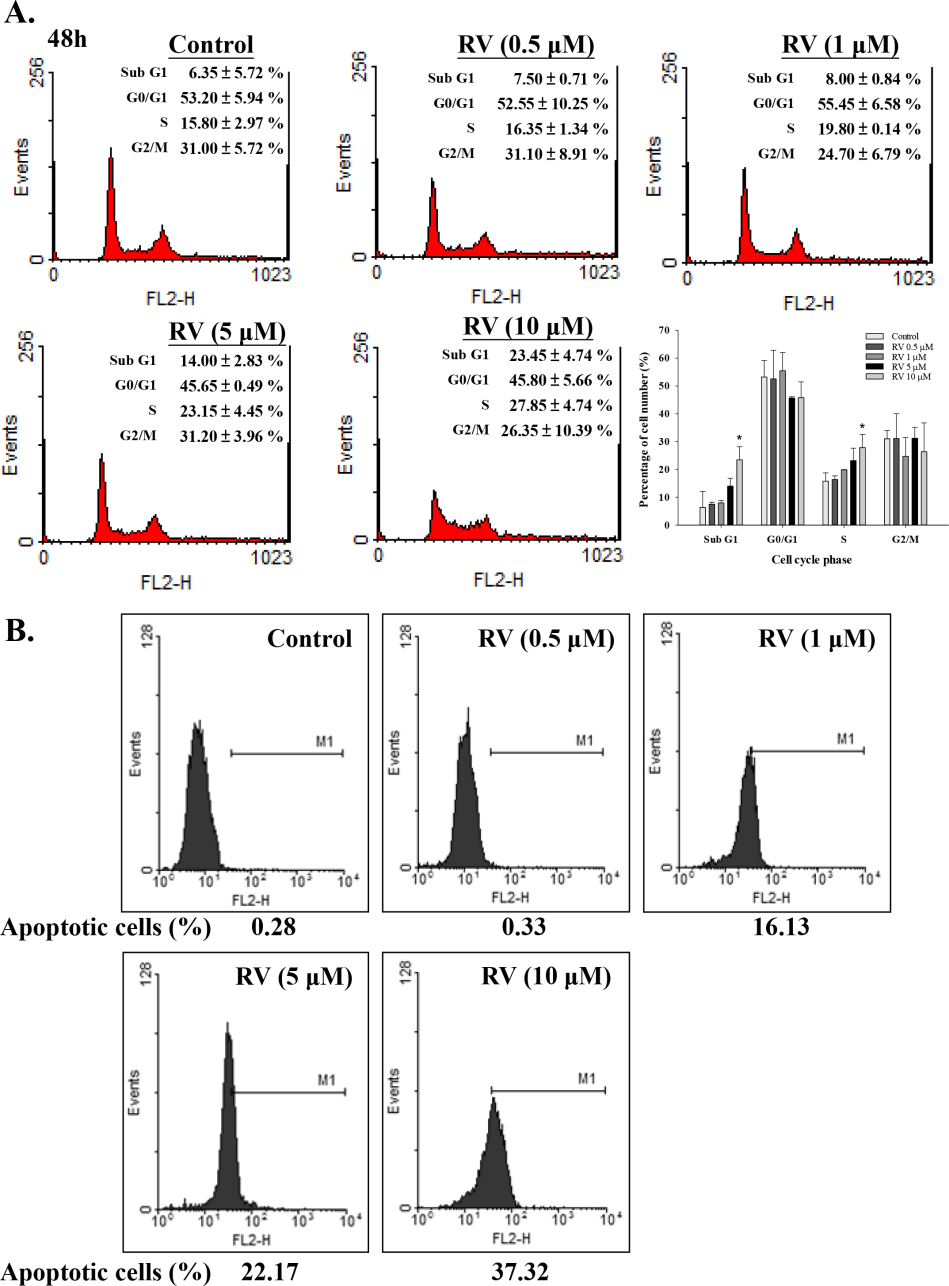

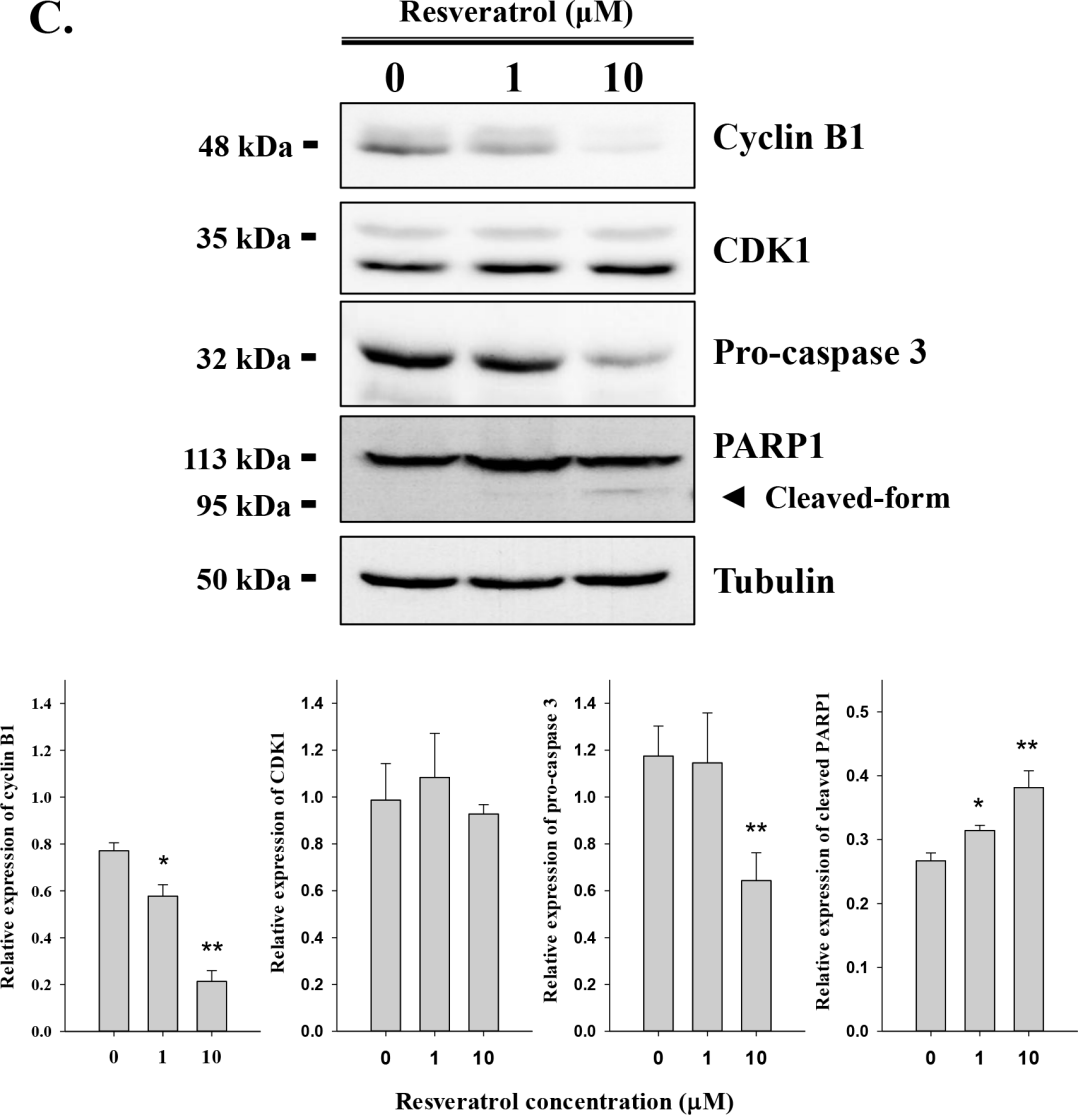
The effects of resveratrol on cell cycle progression in MDA-MB-231 cells **(A)** MDA-MB-231 cells were treated with different concentrations of resveratrol for 48 h. Cell cycle analysis was conducted as described. N = 2. **(B)** MDA-MB-231 cells were treated with difference concentrations of resveratrol for 24 h. Cells were harvested and TUNEL assay was conducted as described. Results shown were representative data from 3 independent experiments. **(C)** Cells were treated with 0, 1 or 10 μM resveratrol for 48 h. Cell extracts were separated by SDS-PAGE and blotted with anti-cyclin B1, anti-CDK1, anti-pro-caspase 3, and anti-PARP1. Tubulin was used as an internal control. N = 3. (*p < 0.05; **p < 0.005, compared to control with vehicle solvent)

In order to determine the effect of resveratrol-induced apoptosis and cell cycle arrest, cells were treated with different concentration of resveratrol for 24 h. Cells were harvested and flow cytometry was conducted. Resveratrol increased the fraction of cells in S phase from 15.80 ± 2.97% in control to 27.85 ± 4.74% at the 48 h time point in a concentration-dependent manner (Fig. 5A). Cells in G_1_ and G_2_/M phases were slightly decreased after resveratrol treatment indicating that resveratrol may induce S phase growth arrest in MDA-MB-231 cells. Resveratrol also induced apoptosis in a concentration-dependent manner (Fig. 5A and B), which may result from modulation of cell cycle progression. Resveratrol inhibited MDA-MB-231 cell proliferation and increased the fraction of cells in sub-G_1_ by 23.45 ± 4.74%, compared to untreated control. Furthermore, the effects of resveratrol on cyclin B1, CDK1, caspase 3, and PARP1 were also addressed. Results indicated that 10 μM resveratrol significantly inhibited cyclin B1 expression, decreased the content of caspase 3, and activated PARP1 (Fig. 5C).

## DISCUSSION

This study investigated effects of resveratrol-induced anti-proliferation in triple-negative MDA-MB-231 breast cancer cells, but the results shown may be applicable to other cancer cells. Included were studies of changes in the cell microenvironment to determine whether such changes altered drug activity.

When low concentrations of resveratrol were used in the current studies, the efflux concentrations of resveratrol were far below the projected concentrations (Fig. 4C); this is consistent with the short biological half-life of the agent described by others [27]. Studies have indicated that resveratrol in double-distilled water is stable for up to 8 h; on the other hand, resveratrol in incubation medium containing 10% FBS decays after 2-3 h and only 10% of the activity persists at 8 h at 37° (HY Lin *et al*., unpublished observation). Our earlier description of a receptor for resveratrol on integrin αvβ3 [6] on the cancer cell surface raised the possibility investigated here that rapid cellular uptake and metabolism of the stilbene need not be relevant to the pro-apoptotic activity that can be initiated by extracellular resveratrol.

Resveratrol (10 μM) reduced the percentage of proliferating cells to 33% of the control levels after 48 h in perfusion bellows culture system (Fig. 4B). Resveratrol inhibited proliferation and induced apoptosis in human epidermoid carcinoma A431 cells in a dose- and time-dependent manner which was associated with a reduced level of expression of cyclins D1, D2 and E2. Also reduced were levels of CDK2, CDK4 and CDK6, but levels of Cip1/p21 and Kip1/p27 proteins were increased [28]. Wolter et al. have shown down-regulation of the cyclin D1/CDK4 complex by resveratrol in colon cancer cell lines [29]. Benitez *et al*. observed that treatment of LNCaP and PC-3 cells with resveratrol induces apoptosis and that this is associated with the reduced levels of expression of cyclins D1 and E and CDK4, as well as a reduction in cyclin D1/CDK4 kinase activity [30]. Several reports indicate that resveratrol inhibits proliferation of cancer cells by inhibiting cell-cycle progression [28, 31–33]. Kuwajerwala *et al*. have reported that treatment of prostate LNCaP cells with resveratrol causes cells to enter into S phase [34]. This complex of properties of the stilbene may all contribute to its anti-proliferative effects.

Resveratrol in a number of studies has been shown to have low bioavailability. Preclinical studies have shown no correlation between the bioavailability of free resveratrol and its chemopreventive or chemotherapeutic efficacy. The IC_50_ values for inhibition of cell growth by resveratrol have been reported to be in the range of 5 to 10 μM in preclinical models [5, 6]. Our studies indicate, in contrast, that at concentrations as low as 0.1 μM, resveratrol induced more than a 50% decrease in cancer cell numbers compared to control after day 6 (Fig. 4B). Certain aberrancies were detected, e.g., results at 0.1 μM resveratrol. We know that low concentrations of the stilbene have been shown to inhibit apoptosis [7] and that resveratrol is an ERα partial agonist [35]. In such studies, the possibility exists that low concentrations of resveratrol stimulated cell proliferation, e.g., up to 2-3 d, before the anti-proliferative action of the agent predominated. At low physiologic serum concentrations that are normally achieved by nutritional intake, phytoestrogens are likely to act through modulation of estrogen signaling. However, these lower serum concentrations appear insufficient to inhibit tyrosine kinases or other enzymes that may provide alternative targets of phytoestrogen effects [36].

The para-hydroxy group of trans-resveratrol is more reactive than its meta-hydroxy groups in neutral and acidic solutions; the reactivity of meta-hydroxy groups increases under alkaline conditions. Therefore, trans-resveratrol effectively scavenges free radicals over a wide pH range [37]. In this study, we chose acid, neutral and basic pH conditions that might be achieved in the tumor cell microenvironment *in vivo*. The acidic microenvironment around tumor cells may contribute to chemoresistance of cancer cells and alkalinity supports chemosensitivity. Resveratrol decreases Na^+^/H^+^ exchanger (NHE1) activity by several mechanisms, promoting an increase in pHe and a decrease in pHi [38, 39]. One of the present co-authors and his colleagues have shown in studies of NHE1 that the *intracellular* buffering capacity of cells is up to 1.2 pH units [40] and we extrapolated to the *extracellular* microenvironment to obtain the pH 8.6 value with a presumed decrease in NHE1 activity attributable to resveratrol.

Using cDNA microarray (Fig. 3) and qPCR, we demonstrated alterations in the expression of genes associated with cell cycle arrest and apoptosis as a result of resveratrol treatment in MDA-MB-231 cells under various culture conditions. In the current study, transcription of p53-dependent pro-apoptotic genes including *p53, c-fos, c-jun, p21(waf1/Cip1), PIG3* and *BAD* was induced by resveratrol (Fig. 3C). In our earlier studies with human thyroid cancer cells, we found a remarkable increase in the level of p53 mRNA and protein, in conjunction with an increase in the level of p21 (Waf1/Cip1), after exposure to resveratrol [41]. Studies from other groups indicate that resveratrol decreases expression of genes that support proliferation [42]. Thus, resveratrol induces apoptosis by activating p53 signaling mechanisms [43] and by causing transcription of a number of genes linked to apoptosis. Certainly, more than one pro-apoptotic mechanism is involved in the actions of resveratrol on tumor cells. In this regard, activation of Apaf-1, a novel protein induced by resveratrol, has been reported to participate in the cytochrome-C-dependent activation of caspase 3, which triggers a cascade of events in apoptosis [23]. Studies of Narayanan et al. have shown that treatment of human prostate cancer LNCaP cells with resveratrol induces expression and activation of p300 and p53 [35]. This appears to be relevant to earlier studies of ours that showed induction by resveratrol of acetylation of the carboxyl terminus of p53 [44]; this increases the level of sequence-specific DNA binding of p53.

In summary, using a perfusion bellows culture system, we have studied the effects of drug exposure duration and acid-alkaline conditions on resveratrol-induced anti-proliferation in triple-negative breast cancer MDA-MB-231 cells. The results of microarray, qPCR and biological end-points confirm that resveratrol-induced anti-proliferation in cancer cells is not affected by environmental pH. More importantly, recurrent short-term incubation of tumor cells with resveratrol are shown here to be sufficient to induce anti-proliferation. These observations in this model establish a useful platform for quantitating anti-cancer activity of resveratrol under a variety of conditions.

## MATERIALS AND METHODS

### Cell line

Human ER-negative breast cancer MDA-MB-231 (ATCC® HTB-26^™^) cells were purchased from American Type Culture Collection (ATCC, Manassas, VA, USA) by Bioresource Collection and Research Center (BCRC, Hsinchu, Taiwan). The cell line had been tested and authenticated (including isoenzyme analysis, Mycoplasma test, cytogenetics test, tumorigenic test and receptor expression test) by BCRC. We purchased the cell line from BCRC and passaged it in our laboratory for fewer than 6 months after thrown and maintained for further study in Dulbecco's Modified Eagle Medium (DMEM, Life Technologies Corporation, Carlsbad, CA, USA), supplemented with 10% FBS and incubation conditions were 5% CO_2_ at 37°C.

### Study of pharmacodynamics of resveratrol

The bellows perfusion cell culture system we developed is a disposable bioreactor capable of high density cell culture for studies of anti-cancer drug [45]. Five × 10^7^ cells were seeded on polymer flakes in either non-perfusion or perfusion bellows cell modes and incubated at 37°C overnight. Flakes were harvested, trypsinized and cells were collected. Cell numbers were counted. The numbers of original cells attached to flakes were 0.5 × 10^7^ cells/bottle. Cell cultures were refreshed with 10% stripped FBS-containing medium. Resveratrol was added in the medium bottle to the final concentrations indicated.

### Western blot

To examine the signaling pathway of resveratrol induced anti-proliferation, we performed western blot analyses to quantify the protein expression levels in the total cell lysates of MDA-MB-231 cells that were treated with 10 μM resveratrol in different time or pH conditions. Protein samples were resolved on a 10% sodium dodecyl sulfate polyacrylamide gel. A 20-μg quantity of protein was loaded in each well with 5x sample buffer, and the protein samples were resolved by electrophoresis at 100 V for 2 h. The resolved proteins were transferred from the polyacrylamide gel to Millipore Immobilon-PSQ Transfer PVDF membranes (Millipore, Billerica, MA, USA) with the Trans-Blot® SD Semi-Dry Transfer Cell (Bio-Rad Laboratories, Inc., Hercules, CA, USA). The membranes were blocked with a solution of 2% fetal bovine serum in Tris-buffered saline. The membranes were incubated with primary antibodies to phospho-p44/42 MAPK (pERK1/2), ERK2 (Cell Signaling Technology, Inc., Beverly, MA, USA), cyclin B1, CDK1, caspase 3 and poly ADP-ribose polymerase 1 (PARP1) (GeneTex International Corporation, Hsinchu City, Taiwan), GAPDH (Abcam, Cambridge, MA, USA), at 4°C overnight and washed, and the proteins were detected with HRP-conjugated secondary antibodies and ImmobilonTM western HRP Substrate Luminol Reagent (Millipore). Images of the western blots were visualized and recorded by BioSpectrum® Imaging System (UVP, LLC, Upland, CA, USA).

### Liquid chromatography–tandem mass spectrometry (LC/MS/MS)

In LC/MS/MS experiments, samples (20 μL) were injected onto an HP 1100 series HPLC system (Agilent Technologies, Palo Alto, CA, USA) equipped with a narrow-bore column Zorbax Eclipse XDB-C18 (5 μm, 150 × 2.1 mm; Agilent Technologies). Separation was performed using a mobile phase of 0.1% (v/v) acetic acid (A) and 100% acetonitrile (B), with a linear gradient of 20–60% B over 25 min. Flow rate was maintained at 0.2 ml/min and elution was monitored by a diode array detector (200–600 nm). The LC effluent was then introduced into a turbo ion-spray source on a Q/STAR-XL quadrupole/time-of-flight (TOF) hybrid mass spectrometer (Applied Biosystems, Foster City, CA, USA). Negative ESI mass spectra were acquired over the range from m/z 100 to 400. The electrospray voltage was set at –4.5 kV and the source temperature was maintained at 475°C. CID spectra were acquired using nitrogen as the collision gas under collision energies of 25–55 V. High purity nitrogen gas (99.995%) was used as nebulizer, curtain, and heater and collision gas source.

### TUNEL Assay

MDA-MB-231 cells were grown in 100-mm tissue culture dishes until 80% confluent, treated with resveratrol in the concentrations of 0, 0.5, 1, 5 and 10 μM for 24 h. Apoptotic cells were detected by the terminal deoxynucleotidyl transferase biotin-dUTP nick end labeling (TUNEL) staining using the fluorescence method with the APO-BrdU TUNEL assay kit (Invitrogen) according to manufacturer's instructions. Following the TUNEL procedure, cells were immediately analyzed by flow cytometry. The percentage of apoptotic positive cells was performed with ModFit LT software. Experiments were carried out independently at least twice.

### Flow cytometry analysis

Cells were harvested from polymer flakes by trypsinization, washed with PBS and were resuspended in 200 μL PBS (1 × 10^5^ −1 × 10^6^ cells). To quantify cellular DNA content, cells were permeabilized by fixation with 70% ethanol for 30 min at 4°C. Samples were stored in 70% ethanol at −20°C for up to several weeks prior to propidium iodide (PI) staining and flow cytometric analysis. If cellular DNA quantification needed to be performed on the same day, cells after permeabilation were washed in PBS and resuspended in 500 μL of PBS. DNase-free RNase (2.5 μL) was added to the cell suspension and incubation carried out at 37°C for 1 h; cells were then collected and maintained in the dark at room temperature for 30 min. Flow cytometry was carried out on a FACSCalibur^™^ (Becton Dickinson) instrument, using CellQuest software to determine DNA content. Fluorescence-activated cell sorting (FACS) analysis used Annexin V-FITC and propidium iodide. The relative percentages of cells in G1, S, or G2/M phase were calculated from FL-2 histograms using ModFit LT software.

### Microarray

After a 6-h treatment with 10 μM resveratrol, cells were collected by trypsinization and total RNA was extracted using the RNeasy kit (Qiagen). Amount and quality of the RNA fractions were evaluated by UV spectrophotometry (260 and 280 nm wavelength) followed by examination of the probes by capillary electrophoresis on Agilent Bioanalyzers. GeneChip expression and IVT (*in vitro* transcription) labeling kits (Affymetrix, Santa Clara, CA, USA) were used for the synthesis of cDNA and complementary RNA respectively. Biotin-labeled RNA was fragmented and hybridized on human genome U133 plus 2.0 microarrays (Affymetrix) following the manufacturer's instructions. After hybridization (16 h), the microarrays were processed by automated washing on the Affymetrix Fluidics Station 400. Staining of the hybridized probes was performed with fluorescent streptavidin–phycoerythrin conjugates (1 mg/ml; Invitrogen). The scanning of DNA microarrays was carried out on an Affymetrix laser instrument. Microarray quality assessment, condensing of the probe sets, data normalization, and filtering were conducted (Genedata AG, Basel, Switzerland).

### Quantitative real-time PCR

To examine mRNA expression, we treated cells with vehicle (0 μM resveratrol) or 10 μM resveratrol treatments for 6 h under different pH conditions (pH values of medium were 6.8, 7.5 or 8.6). Total RNA was extracted with TRIsure reagent (Bioline Ltd., London, United Kingdom). To eliminate the contamination of genomic DNA, total RNA was treated with DNase I (Life Technologies Corporation, Carlsbad, CA, USA) before first strand cDNA synthesis, according to the manufacturer's instructions. As described previously [41, 46], with slight modification, 1 μg of DNase I-treated total RNA was reverse-transcribed with Tetro RT enzyme (Bioline Ltd.) into cDNA, and used as the template for real-time PCR reactions and analysis. The real time PCR reactions were performed using Rotor-GeneTM SYBR® Green PCR Kit on Rotor-Gene Q (Qiagen, Hilden, Germany). This involved an initial denaturation at 95°C for 5 min, followed by 40 cycles of denaturing at 95°C for 5 sec and combined annealing/extension at 60°C for 10 sec, as described in the manufacturer's instructions. The primer sequences were shown as follows: human NLR family, pyrin domain containing 1 (NLRP1), forward 5′-CCGCATAGCCGTACCTTCAC-3′ and reverse 5′-CCGATGTCACTCGGGCTATC-3′ (Accession No.: NM_033004.3); human caspase 2, apoptosis-related cysteine peptidase (CASP2), forward 5′-GCATGTACTCCCACCGTTGA-3′ and reverse 5′-GACAGGCGGAGCTTCTTGTA-3′ (Accession No.: NM_032982.3); human LSM4 homolog, U6 small nuclear RNA associated (LSM4), forward 5′-CACCATGCTTCCCTTGTCAC-3′ and reverse 5′-AGCTCACCAGGTGTCCATTG-3′ (Accession No.: NM_012321.4); human solute carrier family 12 (potassium/chloride transporters), member 4 (SLC12A4), forward 5′-TGGTGGAGATGCATAACAGTGA-3′ and reverse 5′-TTGGTCAGTCTCATCTGCCG-3′ (Accession No.: NM_005072.4); Homo sapiens BCL2-associated agonist of cell death (BAD), forward 5′- CTTTAAGAAGGGACTTCCTCGCC-3′ and reverse 5′-AAGTTCCGATCCCACCAGGA-3′ (Accession No.: NM_032989.2); Homo sapiens tumor protein p53 (p53), forward 5′-AAGTCTAGAGCCACCGTCCA-3′ and reverse 5′- CAGTCTGGCTGCCAATCCA-3′ (Accession No.: NM_000546.5); Homo sapiens cyclin-dependent kinase inhibitor 1A (p21), forward 5′- CTGGGGATGTCCGTCAGAAC-3′ and reverse 5′-CATTAGCGCATCACAGTCGC-3′ (Accession No.: BT006719.1); Homo sapiens jun proto-oncogene (c-jun), forward 5′-TGAGTGACCGCGACTTTTCA-3′ and reverse 5′-TTAAGATGCCTCCCGCACTC-3′ (Accession No.: NM_002228.3); Homo sapiens FBJ murine osteosarcoma viral oncogene homolog (c-fos), forward 5′-GGAGAATCCGAAGGGAAAGGA-3′ and reverse 5′- AGTTGGTCTGTCTCCGCTTG-3′ (Accession No.: NM_005252.3); Homo sapiens cyclooxygenase 2 (COX-2), forward 5′-GCCAAGCACTTTTGGTGGAG-3′ and reverse 5′-GGGACAGCCCTTCACGTTAT-3′ (Accession No.: AY462100.1); Homo sapiens proliferating cell nuclear antigen (PCNA), forward 5′-TCTGAGGGCTTCGACACCTA-3′ and reverse 5′-TCATTGCCGGCGCATTTTAG-3′ (Accession No.: BC062439.1); Homo sapiens estrogen receptor 1 (ERα), forward 5′-TCTTGGACAGGAACCAGGGA-3′ and reverse 5′-TGATGTAGCCAGCAGCATGT-3′ (Accession No.: NM_000125.3); human glyceraldehyde-3-phosphate dehydrogenase (GAPDH), forward 5′- TGCCAAATATGATGACATCAAGAA-3′ and reverse 5′- GGAGTGGGTGTCGCTGTTG-3′ (Accession No.:NM_002046). Calculations of relative gene expression (normalized to GAPDH reference gene) were performed according to the ΔΔCT method. Fidelity of the PCR reaction was determined by melting temperature analysis.

### Statistical methods and calculations

Nucleotide densities were measured with a Storm 860 phosphorimager followed by analysis with ImageQuant software (Molecular Dynamics, Sunnyvale, CA). Student's *t test*, with *P* < 0.05 as the threshold for significance, was used to evaluate the significance of resveratrol effects.
